# Failure time regression with continuous informative auxiliary covariates

**DOI:** 10.1186/s40488-015-0026-8

**Published:** 2015-02-20

**Authors:** Lipika Ghosh, Jiancheng Jiang, Yanqing Sun, Haibo Zhou

**Affiliations:** 1 Department of Mathematics and Statistics, University of North Carolina at Charlotte, Charlotte, NC 28223, USA; 2 Department of Biostatistics, University of North Carolina at Chapel Hill, Chapel Hill, NC 27599, USA

**Keywords:** Auxiliary covariates, Censoring, Estimated partial likelihood, Local linear smoothing, Validation

## Abstract

In this paper we use Cox’s regression model to fit failure time data with continuous informative auxiliary variables in the presence of a validation subsample. We first estimate the induced relative risk function by kernel smoothing based on the validation subsample, and then improve the estimation by utilizing the information on the incomplete observations from non-validation subsample and the auxiliary observations from the primary sample. Asymptotic normality of the proposed estimator is derived. The proposed method allows one to robustly model the failure time data with an informative multivariate auxiliary covariate. Comparison of the proposed approach with several existing methods is made via simulations. Two real datasets are analyzed to illustrate the proposed method.

## 1 Introduction

In epidemiologic studies, the exposure variable vector *X* is often too difficult or too expensive to measure on the full cohort, whereas an auxiliary variable vector *W* for *X* can be easily measured for all subjects in the study cohort. For example, in a large scale nutritional study, the PIN Study (Savitz et al. 2001), it would be prohibitively expensive to obtain the exact dietary iron intake on each individual recruited. Instead, a self administered quantitative food questionnaire is conducted on all subjects where a crude assessment of iron intake is obtained. The true exposure, the blood serrum ferritin concentration, is only assayed for a validation set consisting of a small subset of the full study cohort. Although the true covariates are missing for most individuals, the existence of some surrogates or auxiliary measurements conveys information about *X* and serves as common proxy measure. Utilizing the available auxiliary information to improve the efficiency of the effects estimation and in turns to increase the power of the study is critical for the success of the studies. In this paper, we study censored failure time regression with a continuous auxiliary covariate vector.

A variety of authors have contributed their work to this field. Related works include Prentice (1982), Pepe et al. (1989), Lin and Ying (1993), Hughes (1993), Lipsitz and Ibrahim (1996), Zhou and Wang (2000), Fan and Wang (2009), Liu et al. (2010), etc. In particular, Prentice (1982) introduced a partial likelihood estimator based on the induced relative risk function. This method was further developed by Pepe et al. (1989) using parametric modeling. Zhou and Pepe (1995) proposed an estimated partial likelihood method for discrete auxiliary covariates to relax the parametric assumptions on the frequency of events and the underlying distributions of covariates. This method was extended by Zhou and Wang (2000) to deal with continuous auxiliary variables, based on the Nadaraya-Watson kernel smoother method (Nadaraya 1964; Watson, 1964). Fan and Wang (2009), Liu et al. (2010) used the same approach for multivariate failure time data with auxiliary covariates. While Zhou and Wang’s (2000) approach is useful in certain situations, there are some restrictions on it. First, the approach is effective only when the auxiliary variable *W* is of low dimension so that the “curse of dimensionality” in nonparametric smoothing can be avoided. Secondly, it requires that, conditionally on *X*, *W* provides no additional information about the hazard of failure; that is, all of the effects of *W* on failure and censoring are mediated through *X*, which is somewhat restricted since *W* may not be a true surrogate and depends on the failure given *X*.

Further, this method does not fully utilize the observations in the non-validation subsample and hence cannot be efficient in certain situations.

We here propose a new method to deal with the above problems associated with the method in Zhou and Wang (2000). The proposed method allows *W* to be multivariate and to be informative in the sense that, conditional *X*, it may provide additional information on the hazard of failure. We first estimate the induced relative risk function with a kernel smoother based on the validation sample, and then improve the estimation by utilizing the information on the incomplete observations from the non-validation subsample. In addition, the local linear smoother (see for example in Fan and Gijbels 1996) is employed to enhance the performance of the kernel smoother in Zhou and Wang (2000) at the boundary regions. Our method will be expected to improve the efficiency of the estimator of Zhou and Wang (2000) in various situations, for example, when auxiliary variable *W* is informative or not very informative about *X* (see also the simulation results). Asymptotic normality of our estimator is derived.

The proposed methodology can be extended to model multivariate failure time data with auxiliary covariates by following the method in Fan and Wang (2009) or Liu et al. (2010).

The paper is organized as follows. In Section 2, we introduce the hazards models. In Section 3 we introduce our new estimation approach to predicting the induced relative risk for individuals in non-validation subsample based on the kernel smoother. In Section 4 we concentrate on the asymptotic properties of the proposed estimators. We conduct simulations in Section 5 to compare the efficiencies of different estimating methods. In Section 6 we apply the proposed methodology to two real datasets.

## 2 Cox’s proportional hazards models

To facilitate exposition, we here employ the notations in Zhou and Wang (2000). Suppose that there are *n* independent individuals in a study cohort. Let {*X_i_*(*t*), *Z_i_*(*t*)} denote the covariate vector for the *i*th subject at time *t* (*i* = 1, …, *n*). Assume that *X_i_*(·) is only observed in the validation subsample which is chosen at the baseline under the ignorable missing mechanism condition (Rubin 1976). Let *Z_i_*(·) be the remaining covariate vector that is always observed, and *W_i_*(·) the informative auxiliary variables for *X_i_*(·). Let η_*i*_ be an indicator variable with η_*i*_ = 1 if the *i*th individual is in the validation set and 0 if in the nonvalidation set. Put *V* = {*i* : η_*i*_ = 1} and *V̄* = {*i* : η_*i*_ = 0}. We assume that individuals in the validation subsample are randomly selected and hence representative. Then observed data for the *i*th subject is {*S_i_*, δ_*i*_, *Z_i_*(·), *W_i_*(·), *X_i_*(·)} if η_*i*_ = 1, and {*S_i_*, δ_*i*_, *Z_i_*(·), *W_i_*(·)} if η_*i*_ = 0, where *S_i_* is the observed event time for the *i*th subject, which is the minimum of the potential failure time *T_i_* and the censoring time *C_i_*, and δ_*i*_ is the indicator of censoring. We consider the following conditional hazard rate function of failure (Cox 1972)

(2.1) $$\lambda \{t;X_{i}(t),Z_{i}(t)\}\equiv \underset{\Delta t↓0}{\text{lim}}[\frac{1}{\Delta t}\text{Pr}\{t\leq T_{i}<t+\Delta t|T_{i}\geq t,X_{i}(t),Z_{i}(t)\}]\;=\lambda_{0}(t)\text{exp}\{\beta_{1}^{\prime }X_{i}(t)+\beta_{2}^{\prime }Z_{i}(t)\},$$

where λ_0_(·) ≥ 0 is the unspecified base-line hazard and $\beta =(\beta_{1}^{\prime },\beta_{2}^{\prime })\prime$ is the relative risk parameter vector to be estimated.

For model (2.1), the relative risk functions are $\gamma_{i}(\beta ,t)\equiv \text{exp}\{\beta_{1}^{\prime }X_{i}(t)+\beta_{2}^{\prime }Z_{i}(t)\}$, and the partial likelihood function for the parameters β is

(2.2) $$PL(\beta )=\prod_{i\in V\cup \bar{V}}{\left\{\frac{\gamma_{i}(\beta ,S_{i})}{\sum_{j\in ℛ(S_{i})}\gamma_{j}(\beta ,S_{i})}\right\}}^{\delta_{i}},$$

where ℛ(*S_i_*) is the risk set at time *S_i_*. However, for *i* ∈ *V̄*, the true variate *X_i_*(*t*) is not observed, and hence the corresponding relative risk function γ_*i*_(β, *t*) is not available and has to be imputed.

Zhou and Wang (2000) used the conditional expectation

(2.3) $$\text{exp}\{\beta_{2}^{\prime }Z_{i}(t)\}E[\text{exp}\{\beta_{1}^{\prime }X_{i}(t)\}|S_{i}\geq t,Z_{i}(t),W_{i}(t)]$$

for the imputation of γ_*i*_(β, *t*) (*i* ∈ *V̄*). Based on data in *V*, they obtained the Nadaraya-Watson kernel estimator (Nadaraya 1964; Watson 1964) of the above imputation and replaced γ_*i*_(β, *t*) for *i* ∈ *V̄* in (2.2) by the kernel estimator, which leads to the estimated partial likelihood. Under the assumption that *W* is not informative, that is, all of the effects of *W* on failure and censoring are mediated through *X*, so that

$$\lambda \{t;X_{i}(t),Z_{i}(t),W_{i}(t)\}\equiv \underset{\Delta t↓0}{\text{lim}}[\frac{1}{\Delta t}\text{Pr}\{t\leq T_{i}<t+\Delta t|T_{i}\geq t,X_{i}(t),Z_{i}(t),W_{i}(t)\}]\;=\underset{\Delta t↓0}{\text{lim}}[\frac{1}{\Delta t}\text{Pr}\{t\leq T_{i}<t+\Delta t|T_{i}\geq t,X_{i}(t),Z_{i}(t)\}]\;=\lambda_{0}(t)\text{exp}\{\beta_{1}^{\prime }X_{i}(t)+\beta_{2}^{\prime }Z_{i}(t)\}\;\equiv \lambda \{t;X_{i}(t),Z_{i}(t)\},$$

they derived the consistency and asymptotic normality of the estimation. However, if *W* is informative, their method will generally be biased (see also Section 5). In addition, since this method directly used information in the auxiliary covariate *W* and estimated the conditional expectation (2.3), it may encounter the so-called “curse of dimensionality” if *W* is of higher dimension. For the present study, we propose a new method for imputation of the relative risk function. The information in *W* will be used in a new way. This leads to a new estimated partial likelihood.

## 3 Estimated partial likelihood with a local smoother

In this section, we introduce our method to estimate the parameters in model (2.1) based on maximizing the estimated partial likelihood.

### 3.1 Local smoother for the relative risk function

Instead of (2.3), we use the conditional expectation of γ_*i*_(β, *t*),

$$\phi_{i}(\beta ,t)=\text{exp}\{\beta_{2}^{\prime }Z_{i}(t)E[\text{exp}\{\beta_{1}^{\prime }X_{i}(t)\}|S_{i}\geq t,Z_{i}(t)],$$

as imputation of γ_*i*_(β, *t*). Let *d* be the dimension of *Z* and let

(3.4) $$r_{i}(\beta ,t)=\eta_{i}\gamma_{i}(\beta ,t)+(1-\eta_{i})\phi_{i}(\beta ,t)$$

be the induced risk function. Put $\zeta_{i}(\beta_{1},t)=\text{exp}(\beta_{1}^{\prime }X_{i}(t))$, and ν_*i*_(β_1_, *t*) = *E*[ζ_*i*_(β_1_, *t*)|*S_i_* ≥ *t*, *Z_i_*(*t*)]. To use the partial likelihood (2.2), we need to estimate ϕ_*i*_(β, *t*) or equivalently ν_*i*_(β_1_, *t*) for *i* ∈ *V̄*. Using the local linear smoother (see for example, Fan and Gijbels 1996) leads to the following (functional) estimators of ν_*j*_(β_1_, *t*) for *j* ∈ *V̄*

(3.5) $${\hat{\nu }}_{j}(\beta_{1},t)=\sum_{i\in V}\omega_{i}(t,Z_{j}(t);h)\zeta_{i}(\beta_{1},t),$$

where *h* is the bandwidth,

$$\omega_{i}(t,Z_{j}(t);h)=\frac{\left\{s_{2}-(Z_{i}(t)-Z_{j}(t))s_{1}\}Y_{i}(t)K_{h}(Z_{i}(t)-Z_{j}(t)\right)}{\sum_{i\in V}\{s_{2}-(Z_{i}(t)-Z_{j}(t))s_{1}\}Y_{i}(t)K_{h}(Z_{i}(t)-Z_{j}(t))},$$

*Y_i_*(*t*) = *I*_[*S_i_*≥*t*]_ is the at-risk indicator, *s_k_* = ∑_*i*∈*V*_(*Z_i_*(*t*)−*Z_j_*(*t*))^*k*^*Y_i_*(*t*)*K_h_*(*Z_i_*(*t*)−*Z_j_*(*t*)), and *K_h_*(·) = *h*^−*d*^*K*(·/*h*) for a *d*-variate kernel function *K*(·).

The above estimation of the relative risk function was similarly used in Zhou and Wang (2000) for a nonparametric smoothing problem on the estimation of *E*[γ_*i*_(β, *t*)|*S_i_* ≥ *t*, *Z_i_*(*t*),*W_i_*(*t*)], where the “curse of dimensionality” problem can happen if *W* is multivariate. Note that this estimation method uses only the complete observations in *V* and neglects the important information on incomplete observations in *V̄*. It follows that this approach cannot be expected to be efficient in certain situations. In addition, it is required in Zhou and Wang (2000) that, conditional on *X*, the auxiliary variable *W* provides no additional information on the the hazard of failure. This requirement may not hold if *W* is not a genuine surrogate of *X*. In the following, we propose an improved estimation approach which utilizes information from *W* and observations in *V̄* and does not impose the requirement. Moreover, the proposed method allows one to model the failure time data with informative multivariate auxiliary variable *W* without “curse of dimensionality”. Note that even for one dimensional *Z* and *W*, the method in Zhou and Wang (2000) requires a two-dimensional smoother while the new method needs only one-dimensional smoothing. To have a performance comparable with that of a one-dimensional nonparametric smoother using *M*_1_ = 50 data points, we need about $M=M_{1}^{1.2}=109$ data points for a 2-dimensional nonparameteric smoother. Hence the loss of efficiency due to highly dimensional smoothing is large and increasing exponentially fast (see page 317 of Fan and Yao 2003).

### 3.2 Improved estimation of the relative risk function and the estimated partial likelihood

Recall that *W* is a vector of auxiliary variables for *X* and is hence correlated with *X*. Let ξ_*i*_(α, *t*) = exp(α′*W_i_*(*t*)), where α is a parameter vector to be chosen. Considering the conditional expectation of ψ_*i*_(α, *t*) = *E*[ξ_*i*_(α, *t*)|*S_i_* ≥ *t*, *Z_i_*(*t*)], then we can estimate ψ_*i*_(α, *t*) by running local linear smoothing based on the data in *V*:

(3.6) $${\hat{\psi }}_{j}(\alpha ,t)=\sum_{i\in V}\omega_{i}(t,Z_{j}(t);h)\xi_{i}(\alpha ,t).$$

The following result depicts asymptotic correlation of ν̂_*j*_(β_1_, *t*) and ψ̂_*j*_(α, *t*).

#### Proposition 3.1

*Suppose that the conditions in* Appendix 1 *hold. Given* (*S_j_* ≥ *t*, *Z_j_*(*t*)), $\sqrt{nh^{d}}[({\hat{\nu }}_{j}(\beta_{1},t)-\nu_{j}(\beta_{1},t)),({\hat{\psi }}_{j}(\alpha ,t)-\psi_{j}(\alpha ,t))]$ *is jointly asymptotically normal with mean zero and covariance matrix*

$$\Sigma =\nu_{0}(K)p^{-1}(Z_{j})[\begin{array}{cc} \sigma_{1}^{2}(Z_{j},t) & \rho_{\alpha }^{*}(Z_{j},t)\sigma_{1}(Z_{j},t)\sigma_{2}(Z_{j},t)\\ \rho_{\alpha }^{*}(Z_{j},t)\sigma_{1}(Z_{j},t)\sigma_{2}(Z_{j},t) & \sigma_{2}^{2}(Z_{j},t) \end{array}],$$

*where* ν_0_(*K*) = ∫ *K*^2^(*u*)*du*, $\sigma_{1}^{2}(Z_{j},t)=\text{Var}[\zeta_{j}|S_{j}\geq t,Z_{j}],\sigma_{2}^{2}(Z_{j},t)=\text{Var}[\xi_{j}|S_{j}\geq t,Z_{j}]$, *and p*(·) *is the density function of Z*.

By the distribution theory for multivariate normal variates, the conditional distribution of $\sqrt{nh^{d}}[{\hat{\nu }}_{j}(\beta_{1},t)-\nu_{j}(\beta_{1},t)]$ given $\sqrt{nh^{d}}[{\hat{\psi }}_{j}(\alpha ,t)-\psi_{j}(\alpha ,t)]$ is asymptotically normal with mean

$$\rho_{\alpha }^{*}(Z_{j},t)\frac{\sigma_{1}(Z_{j},t)}{\sigma_{2}(Z_{j},t)}\sqrt{nh^{d}}[{\hat{\psi }}_{j}(\alpha ,t)-\psi_{j}(\alpha ,t)].$$

The conditional mean can be estimated by substituting consistent estimators based on the validation sample for $\rho_{\alpha }^{*}(Z_{j},t)$, σ_1_(*Z_j_*, *t*) and σ_2_(*Z_j_*, *t*), and by replacing ψ_*j*_(α, *t*) with the primary sample based estimator

(3.7) $${\bar{\psi }}_{j}(\alpha ,t)=\sum_{i\in V\cup \bar{V}}{\bar{\omega }}_{i}(t,Z_{j}(t);h)\xi_{i}(\alpha ,t),$$

where

$${\bar{\omega }}_{i}(t,Z_{j}(t);h)=\frac{\left\{{\bar{s}}_{2}-(Z_{i}(t)-Z_{j}(t)){\bar{s}}_{1}\}Y_{i}(t)K_{h}(Z_{i}(t)-Z_{j}(t)\right)}{\sum_{i\in V\cup \bar{V}}\{{\bar{s}}_{2}-(Z_{i}(t)-Z_{j}(t)){\bar{s}}_{1}\}Y_{i}(t)K_{h}(Z_{i}(t)-Z_{j}(t))}$$

and *s̄_k_* = ∑_*i*∈*V*∪*V̄*_ (*Z_i_*(*t*) − *Z_j_*(*t*))^*k*^ *Y_i_*(*t*)*K_h_* (*Z_i_*(*t*) − *Z_j_*(*t*)).

By equating $\sqrt{n_{\nu }h^{d}}[{\hat{\nu }}_{j}(\beta_{1},t)-\nu_{j}(\beta_{1},t)]$ with its estimated conditional mean and solving for ν_*j*_(β_1_, *t*), we obtain an improved (functional) estimate

(3.8) $${\bar{\nu }}_{j}(\beta_{1},t)={\hat{\nu }}_{j}(\beta_{1},t)-{\hat{\rho }}_{\alpha }^{*}(Z_{j},t)\frac{{\hat{\sigma }}_{1}(Z_{j},t)}{{\hat{\sigma }}_{2}(Z_{j},t)}[{\hat{\psi }}_{j}(\alpha ,t)-{\bar{\psi }}_{j}(\alpha ,t)].$$

The updated estimator ν̄_*j*_(β_1_, *t*) is doomed to be more accurate than ν̂_*j*_(β_1_, *t*) in (3.5), since it has used the information from *W* and observations in *V̄*. Even though the information about *W* may not be utilized in a very efficient way as in Zhou and Wang’s (2000) estimator when *W* is not informative, it is the price we have to pay for achieving robustness against informative *W*. Note that ν̄_*j*_ depends on α which is related to efficiency of the estimator. Intuitively, one should choose α to maximize the conditional correlation coefficient between ζ_*j*_ and ξ_*j*_, given (*S_j_* ≥ *t*, *Z_j_*), which is evident from the following result.

#### Proposition 3.2

*Assume that the conditions in* Appendix 1 *hold. Given* (*S_j_* ≥ *t*, *Z_j_*(*t*)), *then we have*

$$\sqrt{nh^{d}}[{\bar{\nu }}_{j}(\beta_{1},t)-\nu_{j}(\beta_{1},t)\overset{𝒟}{\to }𝒩(0,\Omega ),$$

*where* $\Omega (Z_{j},t)=\sigma_{1}^{2}(Z_{j},t)[1-(1-\rho )\rho_{\alpha }^{*2}(Z_{j},t)]\nu_{0}(K)p^{-1}(Z_{j})$.

When $\rho_{\alpha }^{*}=0$, i.e. the relative risk contributed by *W* is not correlated to that contributed by *X*, given (*S* ≥ *t*, *Z*), the estimator ν̄_*j*_ is asymptotically equivalent to ν̂_*j*_ in (3.5).

In general, $\rho_{\alpha }^{*}>0$. Hence, by Propositions 3.1 and 3.2, ν̄_*j*_ is more efficient than ν̂_*j*_. Note that the proposed estimator is consistent for any α.

The above estimation method for ν_*j*_(β, *t*) was similarly used in Chen and Chen (2000) for estimating parameters in a parametric regression model. Our estimation can be regarded as an extension of their estimation approach in nonparametric regression. In addition, we do not need a working model to specify the regression relationship between the surrogate and the covariate, and hence there is no risk of misspecification of the working model.

For each given value of β, with the estimator ν̄_*j*_(β, *t*), we can estimate the induced relative risk *r_i_*(β, *t*) in (3.4) by

(3.9) $${\hat{r}}_{i}(\beta ,t)=\eta_{i}\gamma_{i}(\beta ,t)+(1-\eta_{i}){\bar{\phi }}_{i}(\beta ,t),$$

where ${\bar{\phi }}_{i}(\beta ,t)={\bar{\nu }}_{i}(\beta_{1},t)\text{exp}\{\beta_{2}^{\prime }Z_{i}(t)\}$. Then the parameters β can be estimated by maximizing the following estimated partial likelihood function (EPL):

(3.10) $$EPL(\beta )=\prod_{i=1}^{n}{\left\{\frac{{\hat{r}}_{i}(\beta ,S_{i})}{\sum_{j\in ℛ(S_{i})}{\hat{r}}_{j}(\beta ,S_{i})}\right\}}^{\delta_{i}}.$$

We denote β̂_*EPL*_ = arg max_β_ *EPL*(β).

For an extreme case with *W* ≈ *Z*, Zhou and Wang’s imputation for (2.3) approximately becomes ϕ̂_*i*_(β, *t*) = ν̂_*i*_(β, *t*) exp(β′*Z_i_*(*t*)) and uses a two dimensional smoother, which is inferior to the improved estimator ϕ̄_*i*_(β, *t*), and hence by the definition of β̂_*EPL*_, our estimator is superior to Zhou and Wang’s. However, it is generally difficult to compare these two estimators. In our estimation of the induced relative risk, we used an improved estimator ϕ̄_*j*_(β, *t*) for *j* ∈ *V̄*. The “curse of dimensionality” problem in Zhou and Wang (2000) can be avoided for a multivariate *W*. Our approach would at least be useful in cases where the number of variables in *Z* which are correlated with the missing covariate *X* is low, whereas the exposure variables of interest and their auxiliary variables may be multivariate.

An alternative to β̂_*EPL*_ is to maximize (3.10) but with *r̂_i_*(β, *t*) replaced by *r̃_i_*(β, *t*) = η_*i*_γ_*i*_(β, *t*)+(1−η_*i*_) ϕ̂_*i*_(β, *t*), where ϕ̂_*i*_(β, *t*) = ν̂_*i*_(β, *t*) exp(β′*Z_i_*(*t*)). We denote the resulting estimator by β̂_*V*_, which does not use the information on W in *V̄*. Intuitively, β̂_*EPL*_ should be better than β̂_*V*_, but this is not true in general, since comparison of the asymptotic results in Theorems 4.1 and 4.2 below could not lead to a dominated estimator. However, in small validation ratio settings, β̂_*V*_ is not expected to perform well, since it uses only the observations in the validation set for smoothing.

## 4 Asymptotic behaviors

Let *n_v_* be the subsample size of the validation set and let ρ be the limit of ratio of validation observations, lim_*n*→∞_ *n_v_*/*n*. Assume that ρ ∈ (0, 1]. Define *s*^(0)^(β, *t*) = *E* [*Y_i_*(*t*)*r_i_*(β, *t*)], *s*^(1)^(β, *t*) = (∂/∂β)*s*^(0)^(β, *t*), *s*^(2)^(β, *t*) = (∂/∂β^τ^)*s*^(1)^(β, *t*).

For any matrix *A*, we use *A*^⊗2^ to denote matrix *AA*^τ^.

Let *N_i_*(*t*) = *I*_[*S_i_*<*t*,δ_*i*_=1]_ and

$$M_{i}(t)=N_{i}(t)-\int_{0}^{t}Y_{i}(u)r_{i}(\beta_{0},u)\lambda_{0}(u)du.$$

Define the filters ℱ_*i*_(*t*) = σ {*N_i_*(*u*), *Y_i_*(*u*+), *X_i_*(*u*), *Z_i_*(*u*) : 0 ≤ *u* ≤ *t*}.

The censoring time is assumed to be independent of the failure time conditioning on the true covariates in model (2.1), that is,

(4.11) P{t≤T<t+Δt|T≥t,C≥t,X(t),Z(t)}=P{t≤T<t+Δt|T≥t,X(t),Z(t)},

which is different from that in Zhou and Wang (2000) where it is assumed

P{t≤T<t+Δt|T≥t,C≥t,W(t),Z(t)}=P{t≤T<t+Δt|T≥t,W(t),Z(t)}.

Then, under the independent censoring assumption (4.11),

*M_i_*(*t*) is a mean zero martingale with repsect to ℱ_*i*_(*t*) (Kalbfleisch and Prentice 1980; Fleming and Harrington 1991).

In addition, the cumulative hazard $\Lambda_{0}(t)=\int_{0}^{t}\lambda_{0}(w)dw$ can be consistently estimated as

$${\hat{\Lambda }}_{0}(t)=\int_{0}^{t}{\left[\sum_{i=1}^{n_{\nu }}Y_{i}(u)r_{i}({\hat{\beta }}_{EPL},u)\right]}^{-1}\sum_{i=1}^{n_{\nu }}dN_{i}(u).$$

Without loss of generality, we assume that *t* ∈[0, 1]. Put $\Delta (\phi_{i})(u)=\phi_{i}^{\left(1\right)}(u)/\phi_{i}(u)-s^{\left(1\right)}/s^{\left(0\right)},\Delta (\gamma_{i})(u)=\gamma_{i}^{\left(1\right)}(u)/\gamma_{i}(u)-s^{\left(1\right)}/s^{\left(0\right)},Q_{i}=\int_{0}^{1}\Delta (\phi_{i})(u)Y_{i}(u)[\gamma_{i}(\beta_{0},u)-\phi_{i}(\beta_{0},u)]\lambda_{0}(u)du,Q_{i}^{*}=\int_{0}^{1}\Delta (\phi_{i})(u)Y_{i}(u)\theta_{i}(u;\alpha )\lambda_{0}(u)du$, where $\phi_{1}^{\left(1\right)}(\beta ,u)=(\partial /\partial \beta )r_{i}(\beta ,u)$, and

$$\theta_{i}(u;\alpha )=[\xi_{i}(\alpha ,u)-\psi_{i}(\alpha ,u)]\text{exp}(\beta_{2}^{\prime }Z_{i}(u))\rho_{\alpha }^{*}(Z_{i},u)\sigma_{1}(Z_{i},u)/\sigma_{2}(Z_{i},u).$$

The following theorem shows that β̂_*EPL*_ is asymptotically normal.

### Theorem 4.1

*Suppose that Condition (A) in* Appendix 1 *holds. Then* β̂_*EPL*_ *is consistent estimator for* β *and satisfies*

$$\sqrt{n}({\hat{\beta }}_{EPL}-\beta_{0})\overset{ℒ}{\to }𝒩(0,\Omega ),$$

*where* Ω = *I*^−1^(β_0_)Σ(β_0_)*I*^−1^(β_0_) *with* Σ(β_0_) = (1 − ρ)Σ_1_(β_0_) + ρΣ_2_(β_0_),

$$I(\beta_{0})=-E[\int_{0}^{1}(\frac{r_{i}^{\left(2\right)}(\beta_{0},u)}{r_{i}^{\left(0\right)}(\beta_{0},u)}-{\left\{\frac{r_{i}^{\left(1\right)}(\beta_{0},u)}{r_{i}^{\left(0\right)}(\beta_{0},u)}\right\}}^{⊗2}-\frac{s^{\left(2\right)}(\beta_{0},u)}{s^{\left(0\right)}(\beta_{0},u)}-{\left\{\frac{s^{\left(1\right)}(\beta_{0},u)}{s^{\left(0\right)}(\beta_{0},u)}\right\}}^{⊗2})dN_{i}(t)],$$

$$\Sigma_{1}(\beta_{0})=E{\left[\int_{0}^{1}\Delta (\phi_{i})(u)dM_{i}(u)-(1-\rho )Q_{i}^{*}\right]}^{⊗2},$$

$$\Sigma_{2}(\beta_{0})=E{\left[\int_{0}^{1}\Delta (\gamma_{i})(u)dM_{i}(u)-\frac{1-\rho }{\rho }\{Q_{i}-(1-\rho )Q_{i}^{*}\}\right]}^{⊗2}.$$

### Remark 4.1

It is interesting to note that $Q_{i}^{*}\approx Q_{i}$ when the auxiliary *W* approximates *X*, and hence the second term in the expectation of Σ_2_(β) approximates to (1 − ρ)*Q_i_*. Therefore, a small ρ will not result in a big Σ_2_(β_0_). Theoretically, when *W_i_* = *Z_i_*, $Q_{i}^{*}=0$ and the above asymptotic variance formula shares the same formula as that for the estimator in Zhou and Wang (2000) as exactly expected. However, in practice where *W_i_* ≈ *Z_i_*, since Zhou and Wang (2000) used a higher dimensional smoother than us, our estimator would have better efficiency for finite samples.

When $\rho_{\alpha }^{*}=0$, β̂_*EPL*_ is asymptotically equivalent to the complete-case estimator based on only the validation set *V*. This is also expected, since the auxiliary variable *W_i_* contains no information on *X_i_* at this setting. From Theorem 4.1, the asymptotic covariance matrix of β̂_*EPL*_ is of sandwich form, which can consistently be estimated by Ω̂_0_ = *Î*^−1^(β_0_) Σ̂(β_0_)*Î*^−1^(β_0_), where *Î*(β) and Σ̂(β) are the corresponding empirical estimates. Specifically,

$$Î(\beta )=-n^{-1}\sum_{i=1}^{n}\int_{0}^{1}(\frac{{\hat{r}}_{i}^{\left(2\right)}(\beta ,u)}{{\hat{r}}_{i}^{\left(0\right)}(\beta ,u)}-{\left\{\frac{{\hat{r}}_{i}^{\left(1\right)}(\beta ,u)}{r_{i}^{\left(0\right)}(\beta ,u)}\right\}}^{⊗2}-\frac{{Ŝ}^{\left(2\right)}(\beta ,u)}{{Ŝ}^{\left(0\right)}(\beta ,u)}+{\left\{\frac{{Ŝ}^{\left(1\right)}(\beta ,u)}{{Ŝ}^{\left(0\right)}(\beta ,u)}\right\}}^{⊗2})dN_{i}(t),$$

$${\hat{\Sigma }}_{1}(\beta )={\left(n-n_{\nu }\right)}^{-1}\sum_{i\in \bar{V}}{\left\{\int_{0}^{1}\Delta ({\hat{\phi }}_{i})(t)[dN_{i}(t)-Y_{i}(t){\bar{\phi }}_{i}({\hat{\beta }}_{EPL},t)d{\hat{\Lambda }}_{0}(t)]-(1-\hat{\rho }){\hat{Q}}_{i}^{*}\right\}}^{⊗2},$$

$${\hat{\Sigma }}_{2}(\beta )=n_{\nu }^{-1}\sum_{i\in V}{\left\{\int_{0}^{1}\Delta ({\hat{\gamma }}_{i})(t)[dN_{i}(t)-Y_{i}(t)r_{i}({\hat{\beta }}_{EPL},t)d{\hat{\Lambda }}_{0}(t)]-\frac{1-\hat{\rho }}{\hat{\rho }}[{\hat{Q}}_{i}-(1-\hat{\rho }){\hat{Q}}_{i}^{*}]\right\}}^{⊗2},$$

where ρ̂ = *n_v_*/*n*,

$${\hat{Q}}_{i}=\int_{0}^{1}\Delta ({\hat{\phi }}_{i})(t)Y_{i}(t)[{\hat{r}}_{i}(\beta ,t)-{\hat{\phi }}_{i}(\beta ,t)]d{\hat{\Lambda }}_{0}(t),$$

$${\hat{Q}}_{i}^{*}=\int_{0}^{1}\Delta ({\hat{\phi }}_{i})(t)Y_{i}(t){\hat{\theta }}_{i}(t;\alpha )d{\hat{\Lambda }}_{0}(t),$$

$$\Delta ({\hat{\phi }}_{i})(t)={\hat{\phi }}_{i}^{\left(1\right)}(\beta ,t)/{\hat{\phi }}_{i}(\beta ,t)-{Ŝ}^{\left(1\right)}(\beta ,t)/{Ŝ}^{\left(0\right)}(\beta ,t),$$

$$\Delta ({\hat{\gamma }}_{i})(t)={\hat{\gamma }}_{i}^{\left(1\right)}(\beta ,t)/{\hat{\gamma }}_{i}(\beta ,t)-{Ŝ}^{\left(1\right)}(\beta ,t)/{Ŝ}^{\left(0\right)}(\beta ,t),$$

$${\hat{\theta }}_{i}(t;\alpha )=[\xi_{i}(\alpha ,t)-{\bar{\psi }}_{i}(\alpha ,t)]\text{exp}({\hat{\beta }}_{2}^{\tau }Z_{i}(t)){\hat{\rho }}_{\alpha }^{*}(Z_{i},t){\hat{\sigma }}_{1}(Z_{i},u)/{\hat{\sigma }}_{2}(Z_{i},t).$$

In summary, a constant variance estimator for β̂_*EPL*_ can be obtained by replacing the population quantities in the asymptotic covariance matrix Σ(β_0_) with their corresponding sample averages as in Zhou and Wang (2000). Hence, the asymptotic confidence intervals for β can also be constructed.

The following theorem demonstrates the asymptotic normality of β̂_*V*_.

### Theorem 4.2

*Under the same conditions as in Theorem 4.1, the estimator* β̂_*V*_ *shares the same asymptotic distribution as* β̂_*EPL*_ *but with* Σ_1_(β_0_) *and* Σ_2_(β_0_) *replaced by* Σ_1__*V*_ (β_0_) *and* Σ_2__*V*_ (β_0_), *respectively, where* $\Sigma_{1V}(\beta_{0})=E{\left[\int_{0}^{1}\Delta (\phi_{i})(u)dM_{i}(u)\right]}^{⊗2}$, *and* $\Sigma_{2V}(\beta_{0})=E{\left[\int_{0}^{1}\Delta (\gamma_{i})(u)dM_{i}(u)-\frac{1-\rho }{\rho }Q_{i}\right]}^{⊗2}$.

### 4.1 Choice of the parameter vector α

The choice of α affects efficiency of β̂_*EPL*_, although the estimator is $\sqrt{n}$-consistent for any α. In this paper, we choose α by minimizing the variance of the estimator β̂_*EPL*_. Given initial value of β and α, one can estimate α by minimizing the trace of Ω̂(α).

Once the value of α is known, maximization of *EPL*(β) can be solved via Newton-Raphson iterations. Repeating this procedure, one can find a solution to the optimization problem (3.10). To reduce the burden of computation in practice, one can employ a consistent naive estimator of β as initial value, for example the estimator of β based on only the validation sample which is easy to implement because it involves only a simple fit for the usual Cox’s model. In our experience, using the naive estimator as the initial value the iterations converge in a few steps.

### 4.2 Choice of the bandwidth parameter

As for the bandwidths, they affect the estimator β̂_*EPL*_, which is true in any nonparametric smoothing problems. Fortunately, the proposed estimator β̂_*EPL*_ is effective for a large range of bandwidths (see Condition (6) in Appendix 1). Similar to that in Zhou and Wang (2000), we employed here the empirical bandwidth *h* = (*h*_1_, *h*_2_)′ with *h*_1_ = 2σ̂_*Z*_*n*^−1/3^ and *h*_2_ = 2σ̂_*W*_*n*^−1/3^, where σ̂_*Z*_ and σ̂_*W*_ are respectively the sample standard deviations of *Z* and *W*, which satisfy the bandwidth conditions required in this paper.

## 5 Simulations

In this section, we conduct finite-sample simulations^a^ The aims of the simulations are three-fold: one is to examine the small sample behavior of β̂_*EPL*_, another is to compare the performance of our estimator with some existing estimators under various situations, and the third and the most important is to illustrate that the proposed estimation allows for an informative auxiliary vector *W*. The covariates (*X*, *Z*) are generated from the following transformation to create correlation:

(5.12) $$(\begin{array}{c} X\\ Z \end{array})=(\begin{array}{cc} 1 & 0.0\\ 0.5 & 1 \end{array})(\begin{array}{c} U_{1}\\ U_{2} \end{array}),$$

where *U_i_*’s are independent and identically distributed as *U*(0, 2). The failure time *T* conditional on covariate *X* is from an exponential distribution with hazard function

$$\lambda (t;X)=\lambda \text{exp}(\beta_{1}X+\beta_{2}Z),$$

where λ is the baseline constant hazard. We only consider the case λ = 1. Then

$$f(t;X,Z)=\text{exp}(\beta_{1}X+\beta_{2}Z)\text{exp}(-t\text{exp}(\beta_{1}X+\beta_{2}Z)).$$

The auxiliary variable *W* is generated from

(5.13) W=X+γlog(T)+e,

where *e* ~ 𝒩(0, σ^2^) and σ^2^ is the parameter controlling the strength of the association between *X* and *W*. We consider the settings with γ = 0 and 2. Model (5.13) with γ = 2 allows one to explore the effectiveness of the proposed method with an informative surrogate *W*. For γ = 0, it also allows us to compare the performance of the newly proposed method and that in Zhou and Wang (2000). We do simulations for σ = 0.2 and 0.8. The censoring variable is uniformly distributed and independent of the failure time. The validation set is randomly selected with *P*(η_*i*_ = 1) = 0.5.

We choose the Gaussian kernel function with the bandwidths (*h*_1_ = 2σ̂_*Z*_*n*^−1/3^, *h*_2_ = 2σ̂_*W*_*n*^−1/3^) which satisfy the bandwidth conditions in Theorem 4.1, where σ̂_*Z*_ and σ̂_*W*_ are the sample standard deviations of *Z* and *W* respectively. In the following tables, β_0_ =[log(2), 0.5]′ denotes the true value of the parameter to be estimated, *se* is the standard error of β̂_*EPL*_ from simulation, $\text{mean}(\hat{se})$ denotes the mean of the estimated standard errors and *cp* denotes the 95% coverage probability.

The methods we considered are the newly proposed estimated partial likehood estimation (β̂_*EPL*_)and its conterpart (β̂_*ZW*_) in Zhou and Wang (2000), the estimator (β̂_*V*_) which does not use the information on W in *V̄*, the complete-case Cox regression analysis (β̂_*CC*_) which uses only the validation subsample, the Cox regression with *W* substituted for the missing *X* (β̂_*N*_), and the full data Cox regression (β̂_*F*_) which assumes that *X* is available for all *n* subjects in the study.

Tables 1 and 2 summarizes the results obtained from the simulation. Note that β̂_*F*_, β̂_*CC*_ and β̂_*V*_ in Table 2 are the same in Table 1, so we do not report them in Table 2. For finite sample sizes, the mean, median, standard error (*se*) and 95% confidence intervals of the estimator are calculated based on 500 independent runs. We observe that β̂_*F*_ is the best estimator but it is not always obtainable for practical studies. The estimator β̂_*N*_ is biased. In all the situations β̂_*EPL*_ is observed to be a consistent estimator of true β_0_. The estimates obtained from Zhou and Wang (2000) method are biased when γ = 2. Also, we notice that the bias in their estimates increases when σ increases. Efficiency of β̂_*EPL*_ relative to the complete case estimator β̂_*CC*_ is approximately the same for β_1_ = log(2) but much higher for β_2_ = 0.5. For γ = 0 the estimator β̂_*ZW*_ is more efficient than the proposed estimator for smaller values of σ but as the correlation between the exposure and auxiliary variable decreases the efficiency becomes closer (see the values of *se* for *n* = 300). Also, we notice that β̂_*EPL*_ has less bias than β̂_*V*_ for different values of *n*, but they are still comparable and even in some cases β̂_*V*_ is better in terms of the standard deviation. For γ = 2, our method stays almost equally efficient as σ increases, but β̂_*ZW*_ fails because of its large bias and low coverage probability (*cp*). Note that, when γ = 2, *W* is an informative auxiliary variable about the failure time and is not very informative about *X*.

We also performed simulations to see the effect of validation ratio and different bandwidths on the estimation. The proposed estimator β̂_*EPL*_ works well for smaller validation percentages and is not very sensitive to the bandwidth selection. In particular, β̂_*EPL*_ is better than β̂_*V*_ when the validation ratio is 0.25, which is evidenced in Table 3. We also conducted simulations with smaller validation ratios. Our experience indicates that, as the validation ratio gets as small as 0.2, β̂_*V*_ is very bad but β̂_*EPL*_ still works.

We conclude that, the proposed partial likelihood estimator can be used to make inference for β under various situations. In particular, the estimator is consistent and efficient when the auxiliary variable is informative about the hazard rate of failure time while Zhou and Wang’s estimator fails.

## 6 Real data analysis

### 6.1 Primary Biliary Cirrhosis data

We apply the proposed approach to the data from the Mayo Clinic trial in primary biliary cirrhosis (PBC) of the liver conducted between 1974 and 1984. A total of 424 PBC patients, referred to Mayo Clinic during that ten-year interval, met eligibility criteria for the randomized placebo controlled trial of the drug D-penicillamine. The first 312 cases in the data set participated in the randomized trial and contain largely complete data. The additional 112 cases did not participate in the clinical trial, but agreed to have basic measurements recorded and to be followed for survival. Six of those cases were lost to follow-up shortly after diagnosis, so the data here are on an additional 106 cases as well as the 312 randomized participants.

A clinical background description and a more extended discussion for the trial and the covariates recorded can be found in Dickson et al. (1989) and Markus et al. (1989). The variables involved in our specify analysis include id: case number; days: number of days between registration and the earlier of death, transplantation, or study analysis time; status: status of censoring; bili: serum bilirubin (in mg/dl); chol: serum cholesterol (inmg/dl) and Age: age in days.

In this analysis, we are particularly interested in the effect of patients’ serum cholesterol and age on the survival of the patients. This type of failure time data can be modeled by the Cox proportional hazards models with an unknown baseline hazard function. However, about 31% outcomes of cholesterol were missing in this data set. Removing those observations may lead to biased estimates and standard errors. We noted that the outcomes of serum bilirubin were completely obtained with no missing values. Preliminary analysis showed that there is a significant correlation between serum cholesterol and bilirubin. Also, intuitively bilirubin has some additional effect on the hazard of failure and we would like to use that information efficiently. To illustrate this effect, we performed a complete Cox regression analysis for two different situations. We take the logarithmic transformation of bilirubin for our study.

In Table 4, we observe that the coefficient and standard error estimates are quite different for both the situations and the 95% confidence intervals for the coefficient of age are nonoverlapping. We can conclude that serum bilirubin has some additional effect on the hazard of failure. Hence, our proposed method can be applied to this dataset considering serum bilirubin as the informative auxiliary covariate.

Table 5 displays the analysis results based on the Cox’s regression for the complete data (CC), the method proposed by Zhou and Wang (2000) (ZW) and the proposed method (EPL). The CC method uses only 284 complete-case observations and the other two methods use all 418 observations. Variable “logchol” denotes the logarithm of cholesterol. The estimates of the coefficients and their standard errors are given in the table.

The regression analysis confirms that both serum cholesterol and age are significantly related to the time to event. For estimating the effect of serum cholesterol and age, there is a reasonable efficiency gain by using the two methods based on partial likelihood approach over the complete case Cox regression analysis. But there is a discrepancy between the estimates from complete data and Zhou and Wangs estimate which could be due to the fact that the latter method does not consider the additional effect contributed by the auxiliary covariate. In our simulation we observed that the standard error of the estimates were underestimated in Zhou and Wangs method when auxiliary variable was informative. In the real data analysis also the standard error estimate for serum cholesterol is underestimated. Moreover, the standard error estimates in our method is comparable to Zhou and Wangs method whereas the calculation time is much less compared to their method.

### 6.2 Serrum Ferritin Concentration in relation to preterm delivery study

We apply the proposed approach to the data on iron intake in relation to preterm delivery study from the University of North Carolina Hospitals at Chapel Hill. A total of 1520 women were included in the study. 17 of these women were lost to follow up. So the data consist of 1503 individuals among which 270 individuals had their serrum ferritin concentration (*FERRITIN*) measured with an immunometric assay. However a crude score for dietary iron intake (*DTFE*) was collected using a dietary food frequency questionnaire for all the individuals.

A clinical background description and a more extended discussion for the trial and the covariates recorded can be found in Savitz et al. (2001). The variables involved in our specfic analysis include (i) id: case number; (ii) Gestation Time: The number of weeks from pregnancy to delivery; (iii) *DTFE*: Dietary iron intake(in 100mg/dl); (iv) *Ferritin*: Serum Ferritin (in 100 mg/dl); and (v) *Age*: age in years. By using the notations in the proposed method, X is *Ferritin*, W is *DTFE*, and Z is *Age*.

In this analysis, we are particularly interested in the effect of patients’ serum ferritin and age on the delivery of the patients. This type of failure time data can be modeled by the Cox proportional hazards models.

However, outcomes for serum ferritin were missing in this data set. Removing those observations can lead to biased or inefficient estimates. We noted that the outcomes of dietary iron intake were completely obtained with no missing values.

Table 6 displays the analysis results based on the the CC method, the ZW method, and the proposed EPL method. The CC method used only 270 complete-case observations and the other two methods used all 1503 observations.

The regression analysis using the new method confirms that both serum ferritin and age are significantly related to the time to event. For estimating the effect of serum ferritin and age, there is also a reasonable efficiency gain by using the two methods based on partial likelihood approach over the complete case cox regression analysis. The estimate of serrum ferritin is lower by the EPL method. The estimate is significantly different from zero with p-value 0.020. In contrast, the p-value from CC method in estimation of serrum ferritin is 0.06.

## 7 Conclusion

We have introduced an EPL estimation method for Cox’s models with informative auxiliary covariates and established asymptotic normality of our estimator. The proposed proposed methodology allows for multivariate auxiliary covariates *W* without suffering the curse of dimensionality.

We used the same bandwidth as suggested by Zhou and Wang (2000) in our estimation. Though it performs reasonably well, one can develop a bandwidth selection criteria like generalized cross-validation for an improved estimation. It is desirable to increase the efficiency of the estimation. In future, we can consider the optimization of α or introduce some weight structure in the score equation to achieve robustness. Further, it is worthy extending our approach to model multivariate failure time.

## Supplementary Material

Supplmental

## Figures and Tables

**Table 1:** Comparison of simulation results with σ = 0.2 and validation fraction 0.5

*n*		γ = 0						γ = 2		
			
		β̂_*F*_	β̂_*CC*_	β̂_*N*_	β̂_*V*_	β̂_*ZW*_	β̂_*EPL*_	β̂_*N*_	β̂_*ZW*_	β̂_*EPL*_
50% *censoring*										
100	*mean* − β_0_	0.018	0.021	−0.034	−0.036	−0.045	0.006	−0.988	−0.434	0.031
		0.004	0.030	0.022	0.035	0.013	0.006	0.169	0.174	0.010
	*median* − β_0_	0.013	−0.001	−0.045	−0.047	0.033	−0.033	−0.983	−0.484	0.021
		−0.015	0.023	0.002	0.019	−0.001	0.011	0.160	0.172	0.008
	*se*	0.323	0.439	0.302	0.410	0.346	0.457	0.084	0.574	0.459
		0.281	0.391	0.276	0.307	0.283	0.312	0.237	0.324	0.338
	$\text{mean}(\hat{se})$	0.292	0.429	0.280	0.402	0.329	0.414	0.068	0.373	0.411
		0.258	0.382	0.257	0.278	0.257	0.296	0.244	0.274	0.288
	*cp*	0.916	0.956	0.924	0.938	0.948	0.946	0.0	0.670	0.946
		0.930	0.960	0.934	0.922	0.914	0.924	0.920	0.892	0.916
300	*mean* − β_0_	−0.019	0.026	−0.068	−0.039	−0.007	−0.021	−0.994	−0.630	0.005
		0.006	0.017	0.024	0.013	0.010	0.002	0.166	0.202	−0.007
	*median* − β_0_	−0.028	0.024	0.077	−0.042	−0.012	−0.018	−0.991	−0.635	−0.002
		0.007	0.001	0.028	0.021	0.018	0.002	0.169	0.199	0.003
	*se*	0.161	0.234	0.158	0.225	0.176	0.238	0.048	0.246	0.243
		0.146	0.217	0.146	0.162	0.150	0.163	0.127	0.137	0.166
	$\text{mean}(\hat{se})$	0.164	0.233	0.158	0.227	0.177	0.222	0.039	0.170	0.231
		0.146	0.209	0.145	0.159	0.147	0.155	0.137	0.125	0.159
	*cp*	0.944	0.942	0.928	0.948	0.950	0.936	0.0	0.108	0.940
		0.956	0.942	0.956	0.948	0.944	0.938	0.796	0.630	0.940

20% *censoring*										
100	*mean* − β_0_	0.021	0.020	−0.031	−0.041	0.044	0.002	−1.003	−0.455	0.023
		0.001	0.014	0.018	0.036	0.013	0.005	0.155	0.163	0.011
	*median* − β_0_	0.016	0.014	−0.029	−0.048	0.038	−0.013	−1.000	−0.466	0.003
		−0.008	−0.001	0.011	0.029	0.005	0.003	0.151	0.159	0.005
	*se*	0.248	0.339	0.234	0.322	0.272	0.364	0.071	0.467	0.360
		0.211	0.305	0.210	0.224	0.212	0.229	0.180	0.241	0.235
	$\text{mean}(\hat{se})$	0.232	0.340	0.223	0.306	0.263	0.313	0.062	0.318	0.315
		0.205	0.302	0.204	0.217	0.204	0.213	0.195	0.214	0.215
	*cp*	0.936	0.956	0.934	0.912	0.966	0.894	0.0	0.550	0.904
		0.938	0.956	0.938	0.934	0.952	0.936	0.924	0.862	0.928
300	*mean* − β_0_	−0.007	−0.009	−0.056	−0.032	−0.006	−0.011	−1.001	−0.617	0.015
		−0.001	0.008	0.016	0.008	0.004	−0.004	0.152	0.023	−0.012
	*median* − β_0_	−0.019	−0.016	−0.064	−0.040	−0.006	−0.022	−1.001	−0.613	0.001
		−0.002	0.002	0.011	0.007	0.003	−0.005	0.154	0.024	−0.016
	*se*	0.131	0.190	0.127	0.177	0.141	0.194	0.044	0.304	0.195
		0.116	0.164	0.116	0.126	0.119	0.127	0.100	0.178	0.135
	$\text{mean}(\hat{se})$	0.131	0.187	0.260	0.179	0.142	0.179	0.034	0.219	0.179
		0.116	0.166	0.150	0.125	0.117	0.122	0.110	0.161	0.124
	*cp*	0.948	0.960	0.928	0.952	0.966	0.926	0.0	0.244	0.926
		0.952	0.954	0.952	0.954	0.952	0.944	0.748	0.070	0.934

**Table 2:** Comparison of simulation results with σ = 0.8 and validation fraction 0.5

*n*		γ = 0			γ = 2		
			
		β̂_*N*_	β̂_*ZW*_	β̂_*EPL*_	β̂_*ZW*_	β̂_*ZW*_	β̂_*EPL*_
50% *censoring*							
100	*mean* − β_0_	−0.369	−0.045	0.034	−0.961	−0.325	0.027
		0.139	0.055	0.008	0.177	0.141	0.009
	*median* − β_0_	−0.381	−0.052	0.021	−0.955	−0.368	0.010
		0.028	0.051	0.008	0.164	0.140	0.002
	*se*	0.206	0.374	0.457	0.076	0.549	0.450
		0.260	0.287	0.338	0.243	0.325	0.332
	$\text{mean}(\hat{se})$	0.196	0.256	0.414	0.064	0.374	0.414
		0.249	0.262	0.288	0.244	0.271	.293
	*cp*	0.504	0.940	0.934	0.0	0.721	0.940
		0.914	0.932	0.916	0.904	0.888	0.920
300	*mean* − β_0_	−0.392	−0.056	0.012	−0.965	−0.399	0.012
		0.139	0.033	−0.011	0.175	0.147	−0.009
	*median* − β_0_	−0.392	−0.055	0.004	−0.963	−0.395	0.004
		0.139	0.044	−0.004	0.176	0.145	−0.002
	*se*	0.114	0.198	0.255	0.044	0.325	0.254
		0.142	0.156	0.170	0.129	0.180	0.171
	$\text{mean}(\hat{se})$	0.108	0.223	0.227	0.036	0.213	0.228
		0.140	0.157	0.159	0.137	0.157	0.158
	*cp*	0.068	0.932	0.932	0.0	0.520	0.932
		0.830	0.946	0.934	0.770	0.808	0.936

20% *censoring*							
100	*mean* − β_0_	−0.368	−0.046	0.024	−0.969	−0.328	0.022
		0.126	0.052	0.019	0.163	0.128	0.021
	*median* − β_0_	−0.372	−0.052	0.020	−0.966	−0.354	0.016
		0.122	0.053	0.020	0.168	0.124	0.015
	*se*	0.165	0.272	0.360	0.064	0.450	0.348
		0.202	0.213	0.240	0.186	0.238	0.238
	$\text{mean}(\hat{se})$	0.156	0.263	0.306	0.057	0.291	0.321
		0.198	0.207	0.211	0.195	0.212	0.222
	*cp*	0.352	0.966	0.912	0.0	0.658	0.916
		0.918	0.942	0.928	0.910	0.878	0.924
300	*mean* − β_0_	−0.390	−0.048	0.013	−0.972	−0.388	0.021
		0.123	0.026	−0.011	0.161	0.127	−0.015
	*median* − β_0_	−0.393	−0.058	−0.004	−0.972	−0.399	0.013
		0.123	0.028	−0.017	0.164	0.128	−0.016
	*se*	0.092	0.159	0.195	0.039	0.262	0.200
		0.115	0.123	0.131	0.102	0.138	0.133
	$\text{mean}(\hat{se})$	0.086	0.172	0.186	0.033	0.168	0.179
		0.112	0.123	0.126	0.110	0.122	0.124
	*cp*	0.018	0.954	0.924	0.0	0.412	0.924
		0.792	0.944	0.934	0.716	0.784	0.942

**Table 3:** Comparison of simulation results with β =[*ln*(2) 0.5]′, 50% censoring, σ = 0.2, and validation fraction 0.25

	*n* = 100		*n* = 200	
		
	β̂_*V*_	β̂_*EPL*_	β̂_*V*_	β̂_*EPL*_
*mean* − β_0_	−0.142	0.056	−0.087	0.049
	0.107	0.018	0.056	0.001
*median* − β_0_	−0.152	0.035	−0.125	0.011
	0.091	−0.002	0.065	0.002
*se*	0.506	0.565	0.405	0.417
	0.329	0.320	0.234	0.232
$\text{mean}(\hat{se})$	0.513	0.618	0.380	0.410
	0.306	0.333	0.220	0.224
*cp*	0.944	0.936	0.928	0.924
	0.934	0.954	0.934	0.942

**Table 4:** Regression analysis of primary biliary cirrhosis (PBC) data study

	Method	Variable	Parameter	Standard error	95% Confidence interval
*logbili* < 1.6	CC	logchol	0.271	0.393	(−0.499, 1.040)
		age	0.055	0.012	(0.031, 0.079)

*logbili* ≥ 1.6	ZW	logchol	−0.635	0.345	(−1.312, 0.042)
		age	−0.005	0.016	(−0.037, 0.027)

**Table 5:** Regression analysis of primary biliary cirrhosis (PBC) data

Method	Variable	Estimates of parameters	Standard error	95% Confidence interval
CC	logchol	0.853	0.214	(0.432, 1.273)
	age	0.048	0.010	(0.029, 0.067)

ZW	logchol	1.142	0.154	(0.840, 1.444)
	age	0.047	0.007	(0.033, 0.061)

EPL	logchol	0.851	0.215	(0.429, 1.273)
	age	0.044	0.007	(0.029, 0.058)

**Table 6:** Regression analysis of Iron intake in relation to preterm delivery study

Method	Variables	Estimates of parameters	Standard error	*p*-value	Hazard ratio
CC	ferritin	0.2451	0.1306	0.060	1.278
	age	0.009	0.0108	0.402	1.009

ZW	ferritin	0.2236	0.076	0.004	1.251
	age	0.0102	0.0043	0.018	1.010

EPL	ferritin	0.1797	0.0771	0.020	1.197
	age	0.0159	0.0036	0.000	1.016

## Footnotes

a All numerical results in this paper are obtained using the software MATLAB and the codes are available (Additional file 1).

## Appendix 1: Condition (A)

For the risk function *r_i_*(β, *t*) (as well as for *r̂_i_*(β, *t*), γ_*i*_(β, *t*), ϕ̂_*i*_(β, *t*) and ϕ_*i*_(β, *t*)), we denote by $r_{i}^{\left(j\right)}(\beta ,t)$ the *j*th derivative of *r_i_*(β, *t*) with respect to β, *j* = 0, 1, 2, where $r_{i}^{\left(0\right)}$ means the function itself. Define $S^{\left(0\right)}(\beta ,t)=n^{-1}\sum_{i=1}^{n}Y_{i}(t)r_{i}(\beta ,t)$, *S*^(1)^(β, *t*) = (∂/∂β)*S*^(0)^(β, *t*), *S*^(2)^(β, *t*) = (∂/∂β)*S*^(1)^(β, *t*), *s*^(0)^(β, *t*) = *E*[*Y_i_*(*t*)*r_i_*(β, *t*)], *s*^(1)^(β, *t*) = (∂/∂β)*s*^(0)^(β, *t*), *s*^(2)^(β, *t*) = (∂/∂β)*s*^(1)^(β, *t*). Let $\Delta (\phi )(u)=\frac{\phi^{\left(1\right)}(\beta ,u)}{\phi (\beta ,u)}-\frac{s^{\left(1\right)}(\beta ,u)}{s^{\left(0\right)}(\beta ,u)}$,

$$I(\beta )=-\int_{0}^{1}[\frac{r^{\left(2\right)}(\beta ,u)}{r^{\left(0\right)}(\beta ,u)}-{\left\{\frac{r^{\left(1\right)}(\beta ,u)}{r^{\left(0\right)}(\beta ,u)}\right\}}^{⊗2}-\frac{s^{\left(2\right)}(\beta ,u)}{s^{\left(0\right)}(\beta ,u)}+{\left\{\frac{s^{\left(1\right)}(\beta ,u)}{s^{\left(0\right)}(\beta ,u)}\right\}}^{⊗2}]s^{\left(0\right)}(\beta ,u)dN(u),$$

$$Q=\int_{0}^{1}\Delta (\phi )(u)Y(u)[\gamma (\beta ,u)-\phi (\beta ,u)]\lambda_{0}(u)du,$$

$$Q^{*}=\int_{0}^{1}\Delta (\phi )(u)Y(u)\theta (Z,u;\alpha )\lambda_{0}(u)du.$$

The following conditions are needed for the theoretical results in the paper:

1. $\int_{0}^{1}\lambda_{0}(s)ds<\infty$.
2. *Pr*(*Y*(1) = 1|*V*) > 0 for any validation set *V*.
3. There exists an open subset ℬ, containing the true value β_0_ of β, of the Euclidean space ℛ^*p*^. In addition, $r_{i}^{\left(2\right)}(\beta ,t)$ with elements (∂^2^/∂β_*i*_∂β_*j*_)*r*(β, *t*) exists and is continuous on ℬ for each *t* ∈[0, 1], uniform in *t*, and ϕ(β, *t*) is bounded away from 0 on ℬ×[0, 1]. Furthermore, *I*(β) is positive definite.
4. $$E\{\underset{ℬ\times [0,1]}{\text{sup}}|Y(t)r^{\left(j\right)}(\beta ,t)|\}<\infty ,j=0,1,2,$$
  $$E\{\underset{ℬ\times [0,1]}{\text{sup}}|Y(t){\left(\frac{r^{\left(1\right)}(\beta ,t)}{r(\beta ,t)}\right)}^{⊗2j}r(\beta ,t)|\}<\infty ,j=1,2,$$
  $$E\{\underset{ℬ\times [0,1]}{\text{sup}}|Y(t){\left(\frac{r^{\left(2\right)}(\beta ,t)}{r(\beta ,t)}\right)}^{⊗j}r(\beta ,t)|\}<\infty ,j=1,2.$$
  Also observe that, *s*^(0)^(β, *t*) = *E*[*Y*(*t*)*r*(β, *t*)]= *E*[*Y*(*t*)*r**(β, *t*)].
5. Let *F*_*Y* (*t*),*Z*_ be the joint distribution of (*Y*(*t*), *Z*), and *f* (*t*, *z*) = (∂/∂_*z*_)*F*_*Y* (*t*),*z*_(1, *z*). For each *t* ∈[0, 1], both *f* (*t*, *z*) and ϕ(β, *t*) have the 2nd continuous derivative almost everywhere.
6. *h* → 0, *nh*^*d*+4^ → 0 and *nh^d^*(log *n*)^−2^ → ∞, as *n* → ∞.

## Appendix 2: Technical Proofs

### Proof of Proposition 3.1

The argument employed here is similar to that for Theorem 1 of Jiang et al. (2011). Note that ν̂_*j*_ − ν_*j*_ = ∑_*i*∈*V*_ ω_*i*_(ν_*i*_ − ν_*j*_) + ∑_*i*∈*V*_ ω_*i*_(ζ_*i*_ − ν_*i*_). By standard nonparametric regression techniques (see for example Härdle 1990; Fan and Gijbels 1996), it can be shown that the first term above contributes to bias and is *O_p_*(*h*^2^), which is of order $o_{p}(1/\sqrt{nh^{d}})$, if one uses an undersmoothing bandwidth such that *nh*^*d*+4^ → 0, so that ${\hat{\nu }}_{j}-\nu_{j}=\sum_{i\in V}\omega_{i}(\zeta_{i}-\nu_{i})+o_{p}(1/\sqrt{nh^{d}})$. Similarly, ${\hat{\psi }}_{j}-\psi_{j}=\sum_{i\in V}\omega_{i}(\xi_{i}-\psi_{i})+o_{p}(1/\sqrt{nh^{d}})$. Then the asymptotic normality can be obtained by using the Cramé-Wald device and directly computing the asymptotic mean and variance (see, for example the Lemma 6.3 in Jiang and Mack 2001).

### Proof of Proposition 3.2

Note that from (3.8)

$$\sqrt{nh^{d}}[{\hat{\nu }}_{j}-\nu_{j}]=\sqrt{nh^{d}}[{\hat{\nu }}_{j}-\nu_{j}]\;-\rho^{*}(Z_{j},t)\frac{\sigma_{1}(Z_{j},t)}{\sigma_{2}(Z_{j},t)}\sqrt{nh^{d}}[({\hat{\psi }}_{j}-\psi_{j})+({\bar{\psi }}_{j}-\psi_{j})](1+o_{p}(1)).$$

The asymptotic normality of $\sqrt{nh^{d}}({\bar{\nu }}_{j}-\nu_{j})$ is obtained by the Slutsky’s theorem and the asymptotic normality of $\sqrt{nh^{d}}({\hat{\nu }}_{j}-\nu_{j}),\sqrt{nh^{d}}({\hat{\psi }}_{j}-\psi_{j})$ and $\sqrt{nh^{d}}({\bar{\psi }}_{j}-\psi_{j})$.

#### Lemma 7.1

*Under Condition (A),*

$$\underset{\beta \in ℬ}{\text{sup}}\|n^{-1}\partial Û(\beta ,1)/\partial \beta -(-I(\beta ))\|\overset{P}{\to }0.$$

##### Proof

By simple algebra, we have

$$n^{-1}\partial Û(\beta ,1)/\partial \beta =\int_{0}^{1}n^{-1}\sum_{i=1}^{n}[\frac{{\hat{r}}_{i}^{\left(2\right)}(\beta ,t)}{{\hat{r}}_{i}^{\left(0\right)}(\beta ,t)}-{\left(\frac{{\hat{r}}_{i}^{\left(1\right)}(\beta ,t)}{{\hat{r}}_{i}^{\left(0\right)}(\beta ,t)}\right)}^{⊗2}\;-\frac{{Ŝ}^{\left(2\right)}(\beta ,t)}{{Ŝ}^{\left(0\right)}(\beta ,t)}+{\left(\frac{{Ŝ}^{\left(1\right)}(\beta ,t)}{{Ŝ}^{\left(0\right)}(\beta ,t)}\right)}^{⊗2}]dN_{i}(t).$$

Note that for *j* = 0, …, 2

(7.14) $$\underset{ℬ\times [0,1]}{\text{sup}}\|{\hat{r}}_{i}^{\left(j\right)}(\beta ,t)-r_{i}^{\left(j\right)}(\beta ,t)\|\overset{P}{\to }0,$$

and

(7.15) $$\underset{ℬ\times [0,1]}{\text{sup}}\|{Ŝ}_{i}^{\left(j\right)}(\beta ,t)-s_{i}^{\left(j\right)}(\beta ,t)\|\overset{P}{\to }0,$$

uniformly for *i* = 1, …, *n* if *h* → 0 and *nh^d^* → ∞. It follows that

$$n^{-1}\partial Û(\beta ,1)/\partial \beta =\int_{0}^{1}n^{-1}\sum_{i=1}^{n}[\frac{r_{i}^{\left(2\right)}(\beta ,t)}{r_{i}^{\left(0\right)}(\beta ,t)}-{\left(\frac{r_{i}^{\left(1\right)}(\beta ,t)}{r_{i}^{\left(0\right)}(\beta ,t)}\right)}^{⊗2}\;-\frac{s^{\left(2\right)}(\beta ,t)}{s^{\left(0\right)}(\beta ,t)}+{\left(\frac{s^{\left(1\right)}(\beta ,t)}{s^{\left(0\right)}(\beta ,t)}\right)}^{⊗2}]dN_{i}(t)+o_{p}(1)\;=-I(\beta )+o_{p}(1),$$

uniformly in β ∈ ℬ.

### Proof of Theorem 4.1

The proof is argued in the framework of the multivariate counting processes, the martingale theory, and the techniques commonly used in nonparametric regression. Following the same routine as in Zhou and Wang (2000), the consistency of β̂_*EPL*_ can be derived by using the Inverse Function Theorem (Rudin 1964; Andersen and Gill, 1982) and the argument by Foutz (1977). In the following, we give only the asymptotic normality in Theorem 4.1. The main techniques we employed are Taylor’s expansion of the score function corresponding to the estimated likelihood function (3.10), Lenglart inequality, the martingale central limit theorem (see e.g. Fleming and Harrington 1991), and nonparametric regression techniques.

By using counting process notation, the score function corresponding to the estimated partial likelihood function (3.10) at time point *t* can be written as

(7.16) $$Û(\beta ,t)=\sum_{i=1}^{n}\int_{0}^{t}\Delta ({\hat{r}}_{i})(\beta ,u)dM_{i}(u)+\sum_{i=1}^{n}\int_{0}^{t}\Delta ({\hat{r}}_{i})(\beta ,u)r_{i}(\beta_{0},u)Y_{i}(u)\lambda_{0}(u)du,$$

where

$$\Delta ({\hat{r}}_{i})(u)=\frac{{\hat{r}}_{i}^{\left(1\right)}(\beta ,u)}{{\hat{r}}_{i}(\beta ,u)}-\frac{\sum_{i=1}^{n}Y_{i}(u){\hat{r}}_{i}^{1)}(\beta ,u)}{\sum_{i=1}^{n}Y_{i}(u){\hat{r}}_{i}(\beta ,u)}.$$

By (7.16), β̂_*EPL*_ solves the equation *Û*(β, 1) = 0. By Taylor’s expansion, one gets

(7.17) $$n^{-1/2}Û(\beta ,1)=-n^{-1}\frac{\partial Û(\beta_{*},1)}{\partial \beta }\sqrt{n}({\hat{\beta }}_{EPL}-\beta_{0}),$$

where β_*_ is between β̂_*EPL*_ and β_0_. By Lemma 7.1 and consistency of β̂_*EPL*_,

$$-n^{-1}\frac{\partial Û(\beta_{*},1)}{\partial \beta }\overset{P}{\to }I(\beta_{0}).$$

Therefore, to prove the asymptotic normality in the theorem it suffices to show that *n*^−1/2^*Û* (β, 1) is asymptotically normal with mean 0 and variance Σ(β_0_) = (1−ρ)Σ_1_(β_0_)+ρΣ_2_(β_0_), which is evidenced in Lemma 7.4 below.

### Proof of Theorem 4.2

Using similar arguments to Theorem 4.1, we establish the result.

#### Lemma 7.2

*Under Condition (A)*,

$$n^{-1/2}\sum_{i=1}^{n}\int_{0}^{1}{\left({\hat{r}}_{i}^{\left(k\right)}(\beta ,w)-r_{i}^{\left(k\right)}(\beta ,w)\right)}^{2}Y_{i}(w)r_{i}(\beta ,w)\lambda_{0}(w)dw\overset{p}{\to }0,k=0,1$$

$$n^{-1/2}\sum_{i=1}^{n}\int_{0}^{1}{\left({Ŝ}^{\left(k\right)}(\beta ,w)-S^{\left(k\right)}(\beta ,w)\right)}^{2}Y_{i}(w)r_{i}(\beta ,w)\lambda_{0}(w)dw\overset{p}{\to }0,k=0,1.$$

##### Proof

The result can be obtained by following the same argument as that for Lemma 2.4 of Zhou and Wang (1999).

#### Lemma 7.3

*Under Condition (A), the second term of* *Û* (β, *t*) *in* (7.16) *admits the following decomposition*

$$n^{-1/2}\sum_{i=1}^{n}\int_{0}^{1}\Delta ({\hat{r}}_{i})(\beta ,w)Y_{i}(w)r_{i}(\beta_{0},w)\lambda_{0}(w)dw\;=-n^{-1/2}\sum_{i=1}^{n}\int_{0}^{1}\Delta (r_{i})(\beta ,w)Y_{i}(w)[{\hat{r}}_{i}(\beta ,w)-r_{i}(\beta ,w)]\lambda_{0}(w)dw+o_{p}(1).$$

##### Proof

The proof uses the same argument as that for Lemma 2.5 of Zhou and Wang (1999). By the Taylor expansion

$$f(x,y)=f(x_{0},y_{0})+{\frac{\partial f(x,y)}{\partial x}|}_{x_{0},y_{0}}(x-x_{0})\;+{\frac{\partial f(x,y)}{\partial y}|}_{x_{0},y_{0}}(y-y_{0})+O({\left(x-x_{0}\right)}^{2}+{\left(y-y_{0}\right)}^{2}),$$

if $\frac{\partial^{2}f}{\partial x^{2}},\frac{\partial^{2}f}{\partial y^{2}}$, and $\frac{\partial^{2}f}{\partial x\partial y}$ are finite. Then

$$\frac{{\hat{r}}^{\left(1\right)}}{\hat{r}}=\frac{{\hat{r}}^{\left(1\right)}}{r}-\frac{r^{\left(1\right)}(\hat{r}-r)}{r^{2}}+O[{\left(\hat{r}-r\right)}^{2}+{\left({\hat{r}}^{\left(1\right)}-r^{\left(1\right)}\right)}^{2}]$$

$$\frac{{Ŝ}^{\left(1\right)}}{{Ŝ}^{\left(0\right)}}=\frac{{Ŝ}^{\left(1\right)}}{S^{\left(0\right)}}-\frac{S^{\left(1\right)}(Ŝ-S^{\left(0\right)})}{S^{(0)2}}+O[{\left(Ŝ-S^{\left(0\right)}\right)}^{2}+{\left({Ŝ}^{\left(1\right)}-S^{\left(1\right)}\right)}^{2}].$$

Note that ∑_*i*_ Δ*r̂_i_*(*u*)*r̂_i_*(*u*)*Y_i_*(*u*) = 0. It follows that the left side of the result in the lemma can be expressed as

$$n^{-1/2}\sum_{i=1}^{n}\int_{0}^{1}\Delta ({\hat{r}}_{i})(\beta ,w)Y_{i}(w)r_{i}(\beta ,w)\lambda_{0}(w)dw\;=-n^{-1/2}\sum_{i=1}^{n}\int_{0}^{1}\Delta ({\hat{r}}_{i})(\beta ,w)Y_{i}(w)[{\hat{r}}_{i}(\beta ,w)-r_{i}(\beta ,w)]\lambda_{0}(w)dw\;=-n^{-1/2}\sum_{i=1}^{n}\int_{0}^{1}\Delta (r_{i})(\beta ,w)Y_{i}(w)[{\hat{r}}_{i}(\beta ,w)-r_{i}(\beta ,w)]\lambda_{0}(w)dw+o_{p}(1),$$

where the last equality is from Lemma 7.2. Therefore the result holds.

#### Lemma 7.4

*Under Condition (A)*,

$$n^{-1/2}Û(\beta ,1)\overset{ℒ}{\to }N(0,(1-\rho )\Sigma_{1}(\beta )+\rho \Sigma_{2}(\beta )).$$

##### Proof

Note that *r̂_i_* − *r_i_* = (1 − η_*i*_) (ϕ̄_*i*_ − ϕ_*i*_). Applying the first order approximation $x/y=x_{0}/y_{0}+(x-x_{0})/y_{0}-(y-y_{0})x_{0}/y_{0}^{2}+O({\left(x-x_{0}\right)}^{2}+{\left(y-y_{0}\right)}^{2})$ to *r̂*^(1)^/*r̂* and *Ŝ*^(1)^/*Ŝ*^(0)^ around (*r*^(1)^, *r*) and (*s*^(1)^, *s*^(0)^), respectively, and by Lemma 7.3 the second term of *n*^−1/2^*Û*(β, 1) in (7.16) becomes

(7.18) $$\Delta (r_{i})(\beta ,w)Y_{i}(w)[{\hat{r}}_{i}(\beta ,w)-r_{i}(\beta ,w)]\lambda_{0}(w)dw+o_{p}(1)\;=-n^{-1/2}\sum_{j\in \bar{V}}\int_{0}^{1}({\hat{\phi }}_{j}-\phi_{j})\Delta (\phi_{j})(u)Y_{j}(u)\lambda_{0}(u)du+o_{p}(1)\;=I_{n1}+o_{p}(1).$$

Note that ${\hat{\phi }}_{j}(\beta ,t)={\hat{\nu }}_{j}(\beta_{1},t)\text{exp}\{\beta_{2}^{\prime }Z_{j}(t)\}$. Since

$${\bar{\phi }}_{j}-\phi_{j}=({\hat{\phi }}_{j}-\phi_{j})-\text{exp}\{\beta_{2}^{\prime }Z_{j}(u)\}\rho_{\alpha }^{*}(Z_{j},u)\frac{\sigma_{1}(Z_{j},u)}{\sigma_{2}(Z_{j},u)}({\hat{\psi }}_{j}-{\bar{\psi }}_{j})(1+o_{p}(1))=\;\sum_{i\in V}\omega_{i}(\gamma_{i}-\phi_{j})-\text{exp}\{\beta_{2}^{\prime }Z_{j}(u)\}[\sum_{i\in V}\omega_{i}(\xi_{i}-\psi_{j})\rho_{\alpha }^{*}(Z_{j},u)\frac{\sigma_{1}(Z_{j},u)}{\sigma_{2}(Z_{j},u)}\;-\sum_{i\in V\cup \bar{V}}{\bar{\omega }}_{i}(\xi_{i}-\psi_{j})\rho_{\alpha }^{*}(Z_{j},u)\frac{\sigma_{1}(Z_{j},u)}{\sigma_{2}(Z_{j},u)}](1+o_{p}(1))+o_{p}(\frac{1}{\sqrt{n}})\;=\sum_{i\in V}\omega_{i}[(\gamma_{i}-\phi_{j})-\text{exp}\{\beta_{2}^{\prime }Z_{j}(u)\}\rho_{\alpha }^{*}(Z_{j},u)\frac{\sigma_{1}(Z_{j},u)}{\sigma_{2}(Z_{j},u)}(\xi_{i}-\psi_{j})](1+o_{p}(1))\;+\sum_{i\in V\cup \bar{V}}{\bar{\omega }}_{i}(\xi_{i}-\psi_{j})\text{exp}\{\beta_{2}^{\prime }Z_{j}(u)\}\rho_{\alpha }^{*}(Z_{j},u)\frac{\sigma_{1}(Z_{j},u)}{\sigma_{2}(Z_{j},u)}(1+o_{p}(1))+o_{p}(\frac{1}{\sqrt{n}}),$$

the first term in (7.18) can be rewritten as

$$I_{n1}=-n^{-1/2}\sum_{j\in \bar{V}}\int_{0}^{1}\Delta (\phi_{j})(u)Y_{j}(u)\lambda_{0}(u)\;\times \{\sum_{i\in V}\omega_{i}[(\gamma_{i}-\phi_{j})-\text{exp}\{\beta_{2}^{\prime }Z_{j}(u)\}\rho_{\alpha }^{*}(Z_{j},u)\frac{\sigma_{1}(Z_{j},u)}{\sigma_{2}(Z_{j},u)}(\xi_{i}-\psi_{j})]\;+\sum_{i\in V\cup \bar{V}}{\bar{\omega }}_{i}(\xi_{i}-\psi_{j})\text{exp}\{\beta_{2}^{\prime }Z_{j}(u)\}\rho_{\alpha }^{*}(Z_{j},u)\frac{\sigma_{1}(Z_{j},u)}{\sigma_{2}(Z_{j},u)}\}du(1+o_{p}(1))+o_{p}(1)\;\equiv J_{n1}+J_{n2}+o_{p}(1).$$

Note that

$$n_{\nu }^{-1}\sum_{i\in V}Y_{i}(t)K_{h}(Z_{i}-Z_{j})=f(t,Z_{j})(1+o_{p}(1)),$$

$$n^{-1}\sum_{i\in V\cup \bar{V}}Y_{i}(t)K_{h}(Z_{i}-Z_{j})=f(t,Z_{j})(1+o_{p}(1)),$$

$$\omega_{i}(t,Z_{j};h)=f^{-1}(t,Z_{j})(1+o_{p}(1))n_{\nu }^{-1}Y_{i}(t)K_{h}(Z_{i}-Z_{j}),$$

$${\bar{\omega }}_{i}(t,Z_{j};h)=f^{-1}(t,Z_{j})(1+o_{p}(1))n^{-1}Y_{i}(t)K_{h}(Z_{i}-Z_{j}),$$

uniformly for *j* = 1, …, *n*. Then

$$J_{n1}=-\frac{1}{\sqrt{n}}\sum_{j\in \bar{V}}\int_{0}^{1}\Delta (\phi_{j})(u)Y_{j}(u)\lambda_{0}(u)f^{-1}(u,Z_{j})\times \;\frac{1}{n_{\nu }}\sum_{i\in V}Y_{i}(u)K_{h}(Z_{i}-Z_{j})[(\gamma_{i}-\phi_{j})-\text{exp}\{\beta_{2}^{\prime }Z_{j}(u)\}\;\times \rho_{\alpha }^{*}(Z_{j},u)\frac{\sigma_{1}(Z_{j},u)}{\sigma_{2}(Z_{j},u)}(\xi_{i}-\psi_{j})]du+o_{p}(1)\;=-\frac{1}{\sqrt{n}}\frac{n-n_{\nu }}{n_{\nu }}\sum_{i\in V}[Q_{i}-Q_{i}^{*}]+o_{p}(1),$$

$$J_{n2}=-\frac{1}{\sqrt{n}}\sum_{j\in \bar{V}}\int_{0}^{1}\Delta (\phi_{j})(u)Y_{j}(u)\lambda_{0}(u)\text{exp}\{\beta_{2}^{\prime }Z_{j}(u)\}\rho_{\alpha }^{*}(Z_{j},u)\frac{\sigma_{1}(Z_{j},u)}{\sigma_{2}(Z_{j},u)}\;\times \frac{1}{n}\sum_{i\in V\cup \bar{V}}Y_{i}(u)K_{h}(Z_{i}-Z_{j})(\xi_{i}-\psi_{j})f^{-1}(u,Z_{j})du+o_{p}(1)=\;-\frac{1}{\sqrt{n}}\frac{n-n_{\nu }}{n}\sum_{i\in V\cup \bar{V}}Q_{i}^{*}+o_{p}(1).$$

Therefore, the second term of *n*^−1/2^*Û*(β, 1) in (7.16) equals

$$-\frac{1}{\sqrt{n}}\frac{n-n_{\nu }}{n_{\nu }}\sum_{i\in V}[Q_{i}-Q_{i}^{*}]-\frac{1}{\sqrt{n}}\frac{n-n_{\nu }}{n}\sum_{i\in V\cup \bar{V}}Q_{i}^{*}+o_{p}(1).$$

Hence, *n*^−1/2^*Û*(β, 1) can be expressed as

$$n^{-1/2}\sum_{i\in \bar{V}}[\int_{0}^{1}\{\frac{\phi_{i}^{\left(1\right)}(\beta ,u)}{\phi_{i}(\beta ,u)}-\frac{s^{\left(1\right)}(\beta ,u)}{s^{\left(0\right)}(\beta ,u)}\}dM_{i}(s)-\frac{n-n_{\nu }}{n}Q_{i}^{*}]+o_{p}(1)\;+n^{-1/2}\sum_{i\in V}[\int_{0}^{1}\{\frac{r_{i}^{\left(1\right)}(\beta ,u)}{r_{i}(\beta ,u)}-\frac{s^{\left(1\right)}(\beta ,u)}{s^{\left(0\right)}(\beta ,u)}\}dM_{i}(s)-\frac{n-n_{\nu }}{n_{\nu }}(Q_{i}-Q_{i}^{*}\frac{n-n_{\nu }}{n})].$$

For the 1st and 3rd terms above, each of them is a sum of independently distributed terms with mean zero from the nonvalidation and validation subsamples, respectively. The 1st term converges weakly to a gaussian process with covariance (1−ρ)Σ_1_(β_0_). The 3rd term is asymptotically normal with mean zero and variance ρΣ_2_(β_0_). By independence of the two terms, $n^{-1/2}Û(\beta ,1)\underset{\to }{P}N(0,\Sigma (\beta ))$ with Σ(β) = (1 − ρ)Σ_1_(β) + ρΣ_2_(β).
